# Electroacupuncture Attenuates Fibromyalgia Pain Through Increased PD-1 Expression in Female Mice

**DOI:** 10.3390/brainsci15090976

**Published:** 2025-09-11

**Authors:** I-Han Hsiao, Wei-Hung Chen, Ming-Chia Lin, Hsin-Cheng Hsu, Hsien-Yin Liao, Yi-Wen Lin

**Affiliations:** 1 School of Medicine, College of Medicine, China Medical University, Taichung 404328, Taiwan; 018309@tool.caaumed.org.tw; 2 Department of Neurosurgery, China Medical University Hospital, Taichung 404332, Taiwan; 3 Department of Anesthesiology, E-DA Hospital, I-Shou University, Kaohsiung 82445, Taiwan; 4 Department of Nuclear Medicine, E-DA Hospital, I-Shou University, Kaohsiung 82445, Taiwan; 5 Department of Traditional Chinese Medicine, China Medical University Hsinchu Hospital, Hsinchu 302233, Taiwan; 6 School of Post-Baccalaureate Chinese Medicine, College of Chinese Medicine, China Medical University, Taichung 404328, Taiwan; 7 Graduate Institute of Acupuncture Science, College of Chinese Medicine, China Medical University, Taichung 404328, Taiwan; 8 Chinese Medicine Research Center, China Medical University, Taichung 404328, Taiwan

**Keywords:** acupuncture, PD-L1, TLR4, dorsal root ganglia, microglia, astrocyte

## Abstract

Background/Objectives: Fibromyalgia causes chronic long-term pain, with symptoms lasting for months to years. Given the lack of evidence-based methods for diagnosing and assessing fibromyalgia, it ranks among the most difficult chronic pain conditions to treat. Programmed cell death ligand 1 (PD-L1) can inhibit acute and chronic pain transmission by inhibiting neuronal ion channels. Methods: Here, we aimed to explore the analgesic efficacy and mechanism of PD-L1/PD1 in an intermittent cold stress-induced fibromyalgia pain mouse model. Results: Von Frey and Hargreaves tests were performed, showing that the mouse model exhibited mechanical (day 4: 2.08 ± 0.13 g, *n* = 9) and thermal hyperalgesia (day 4: 3.93 ± 0.45 s, *n* = 9). Electroacupuncture (EA) or intraventricular PD-L1 injection effectively alleviated the nociceptive response and led to low PD-1 levels in the mouse dorsal root ganglia, spinal cord, thalamus, somatosensory cortex, and cerebellum, as measured through Western blots. In contrast, the pain-related kinase levels increased after fibromyalgia induction; these effects were reversed by EA and PD-L1 via the inhibition of microglia/astrocytes and Toll-like receptor 4. Conclusions: Our results show that EA can treat fibromyalgia pain in mice through effects on the PD-L1/PD1 pathway, indicating its potential as a therapeutic target in fibromyalgia.

## 1. Introduction

The prevalence of fibromyalgia pain is predicted to increase owing to the lack of medicines for this symptom, as well as its underlying cause. Fibromyalgia is particularly challenging to treat because the cause of pain is difficult to identify due to incorrect diagnoses, complex symptomatology, and a lack of meaningful biomarkers. So far, there has been no standard therapy or alternative treatments established to help fibromyalgia patients [1]. A recent study suggested that fibromyalgia patients have a predominantly higher salivary cortisol concentration than healthy subjects, which is a risk factor for insomnia, stress, anxiety, and depression [2]. Neuroinflammation has also been linked to fibromyalgia progression [3]. Further, Cordón et al. identified that fibromyalgia patients had fewer inner retinal neurons, suggesting neuroinflammation and neurodegeneration associated with severe fibromyalgia and, thus, a worsened quality of life [4]. In addition, fibromyalgia patients were found to exhibit central sensitization in the brain and spinal cord (SC), with increased pain sensation. A recent article reported that eicosapentaenoic acid can reduce fibromyalgia nociception by moderating microglia/astrocytes and Toll-like receptor 4 in the mice cerebellum 5–7 (CB5-7) [5]. CB5 and 6 are located in the middle of the hemisphere, while further medially is area CB7. Despite medications such as pregabalin, duloxetine, and milnacipran being approved by the US FDA for fibromyalgia pain relief, they have low patient satisfaction and side effects [6]. Fibromyalgia has been estimated to have a higher prevalence in females than in males [7]. Furthermore, a healthy lifestyle including the practice of tai chi, meditation, exercise, or yoga can relax muscles, lower stress, and reduce pain in people with fibromyalgia [8].

Programmed cell death ligand 1 (PD-L1), also known as CD274, regulates cellular immune responses in dendritic cells, lymphocytes, and endothelial cells. It is present in tumor cell membranes and can bind to the programmed cell death protein 1 (PD-1) expressed in T cells. Accordingly, PD-L1/PD-1 signaling modulates immune function within the tumor microenvironment [9]. Chen et al. reported that PD-L1 can inhibit acute and chronic pain by suppressing nociceptive neurons through the PD-1 receptor [10]. Gerdabi et al. reported the appearance of PD-1 in peripheral and central immune cells of individuals with mucoepidermoid carcinoma. There were more peripheral immune cells in those with acinic cell carcinoma than in those with adenoid cystic carcinoma. PD-1 was similarly observed in peripheral and central malignant tumor cells [11]. The relation between PD-L1/PD-1 and pain has been reported, but its association with fibromyalgia is lacking sufficient evidence. A recent article reported that interferon gamma (IFN-γ) significantly induced PD-L1 expression in tumor cells responding to cancer progression [12]. Furthermore, interleukin (IL)-17, IFN-γ, and tumor necrosis factor alpha (TNF-α) can independently control PD-L1 by activating AKT, nuclear factor-κB (NFκB), and extracellular regulated protein kinase (ERK) signaling in cancer cells [13]. In addition, IFN-γ inhibition can attenuate PD-L1 expression via the myeloid differentiation primary response 88 (MyD88)/TNF Receptor-Associated Factor 6 (TRAF6) pathway [14]. Thus, several drugs, including atezolizumab, avelumab, nivolumab, and metformin, aiming to inhibit the PD-L1/PD-1 pathway have been developed for cancer treatment [15].

The communication between neurons and glial cells (mainly microglia (ionized calcium-binding adaptor molecule 1, Iba1-positive) and astrocytes (glial fibrillary acidic protein, GFAP-positive)) in pain signaling is crucial for the development of chronic pain. In case of injury or pathophysiological situations, overactivated microglia, which are considered proinflammatory M1 cells, can further increase the levels of inflammatory cytokines such as ILs, TNF-α, IFN-γ, and chemokines to initiate allodynia and hyperalgesia and cause chronic pain [16,17]. High-mobility group protein B1 (HMGB1) can be released by microglia or astrocytes in response to inflammation and bind to inflammatory receptors such as the receptor for advanced glycation end products and Toll-like receptors (TLR), further activating the mitogen-activated protein kinase and NFκB signaling pathways [18,19]. Astrocytic marker S100 calcium-binding protein B (S100B) has also been indicated to be activated in certain brain areas, resulting in several chronic pain models. TLR4 can participate in innate and adaptive immune responses. It has an extracellular domain to sense extracellular inflammatory mediators such as S100B, HMGB1, and lipopolysaccharide, which can trigger a series of downstream pathways responding to the induction, transduction, and maintenance of chronic pain. Its activation could further trigger MyD88 to increase TRAF6 and mitogen-activated protein kinase (MAPK)/NKκB and the production of proinflammatory cytokines such as IL-1, IL-2, IL-6, TNF-α, and IFN-γ [20,21].

Acupuncture, an ancient form of Chinese medicine, was first used in Asia over 3 thousand years ago for disease treatment, and especially pain management. Given its well-known therapeutic effect as well as minimal side effects, acupuncture is practiced around the world to treat back and dental pain, headaches, tennis elbow, and fibromyalgia. Modern acupuncture involves inserting a very fine steel needle into a specific point on the body called an acupoint; when performed manually, the needle is lifted and rotated to ensure the occurrence of the de-qi, suggesting successful manipulation. Electroacupuncture (EA) is reported to be therapeutically effective and yield consistent results. A recent double-blinded, randomized controlled trial demonstrated that acupuncture could relieve chronic pain and major depressive disorder [22]. Also, it has shown antinociceptive effects in various mouse models of inflammatory and neuropathic pain, as well as fibromyalgia [23,24,25,26]. EA’s effects are considered to occur through increased opioid, dopamine, cannabinoid, and adenosine receptor stimulation. EA can also attenuate the release of proinflammatory IL-1β, IL-6, TNFα, and IFN-γ. In addition, we previously demonstrated that EA could reduce mechanical and thermal hyperalgesia by suppressing transient receptor potential cation channel subfamily V member 1 (TRPV1) signaling [26].

The purpose of this study was to evaluate whether EA could reduce pain associated with fibromyalgia in mice. Our hypothesis was that EA could significantly relieve fibromyalgia pain via PD-1 signaling. We also aimed to elucidate the mechanisms of EA’s alleviation of fibromyalgia pain in detail. We found an association between PD-L1/PD-1 present on microglia/astrocytes and TLR4 and its allied signaling pathways in a mouse model of intermittent cold stress (ICS)-induced fibromyalgia pain. These phenomena were found in mice receiving EA treatment or intracerebroventricular (i.c.v.) PD-L1 injection. Our results provide novel insights into the relationship between EA analgesia and the PD-L1/PD-1 pathway.

## 2. Materials and Methods

### 2.1. Mice and Fibromyalgia Pain Model

The experimental mice were 8–12-week-old female C57BL/6 wild-type mice (18–20 g) bought from BioLasc Taiwan, Ltd. (Yilan, Taiwan). We used female mice to reflect the higher prevalence of fibromyalgia pain in females than in males in humans. After arrival, the mice were placed in a home cage under a 12 h light/dark cycle (light 6 a.m. to 6 p.m.). The temperature was maintained at 25 °C with 60% moisture. The experiments were approved by the Institute of Animal Care and Use Committee of China Medical University (Permit no. CMUI-ACUC-2021-336), Taiwan, according to the Guide for the Care and Use of Laboratory Animal*s* (National Academy Press, Washington, DC, USA). Nine mice per group were considered the minimum number required to detect an effect size of 0.6 with a significance level (alpha) of 0.05 and 80% power [27]. The investigators were blinded with regard to group division and data examination. Mice were randomly divided into four groups: normal (Normal); cold stress-induced fibromyalgia pain (FM); cold stress-induced fibromyalgia pain with EA (FM + EA); and cold stress-induced fibromyalgia pain with i.c.v. PD-L1 injection (FM + PD-L1). To create the mouse FM model, mice were kept in a 4 °C environment, while normal mice were kept at 25 °C. At 10 a.m. the next day, FM mice were transferred to a 25 °C environment for 30 min before being taken back to the 4 °C environment for 30 min. This procedure was repeated until 4 p.m., when they were moved again and kept overnight. This was repeated for the first 3 days.

### 2.2. Electroacupuncture

After being anesthetized with 5% isoflurane for induction, the mice were given 1% isoflurane for maintenance via a head-stuffed tube for inhalation. A pair of 1” acupuncture needles (32G, Yu Kuang Chem. Ind. Corp., Tainan City, Taiwan) was implanted bilaterally into the ST36 acupoint. The ST36 acupoint in mice is 3–4 mm below the patella, between the fibula and tibia, and on the anterior side of the anterior tibial muscle. The acupoint was electrically stimulated with a constant square pulse with 1 mA intensity, 2 Hz frequency, and 100 μs width for 20 min from a Trio 300 stimulator (Ito, Tokyo, Japan). The EA treatment sessions were conducted on day 3 and day 4.

### 2.3. Monitoring of Nociceptive Behavior

In separate Plexiglas boxes perforated overhead, the mice were placed on elevated horizontal wire mesh stands and covered with a dark cloth for 30 min before starting the behavioral test. The environment was kept silent, and room temperature was maintained (25 °C), allowing the mice to habituate. The experiments were only conducted when the mice were calm, with all of their feet placed on a surface, and when they were not grooming or sleeping. A von Frey filament measuring instrument (23924 Victory Blvd., Woodland Hills, CA, USA) was pressed onto the center of the right plantar hind paw of the mice. The maximum pressure was achieved when the right hind paw was lifted using the plastic tip and the mouse reflexively withdrew the hind paw. We allowed for 3 min breaks between stimuli. The results were recorded as mechanical sensitivity. Hargreaves’ test was used for measuring the mice’s thermal sensitivity, and the setup was similar to that used for the von Frey test. The subjects were placed in an animal enclosure that separated the mice to limit interaction and was covered with a dark cloth. After allowing 30 min of habituation, the experiments were initiated. The IITC Plantar Analgesia Meter (23924 Victory Blvd., Woodland Hills, CA, USA) was used to measure the withdrawal latency time of the mice when subjected to radiant heat applied to the center of their right hind paws. A cut-off time of 20 s was set to prevent tissue damage. In the thermal paw withdrawal test, the nociception threshold was assessed using the latency of paw withdrawal upon stimulus and was recorded when the heat applied caused the subject to withdraw its hind paw.

### 2.4. Western Blot Analysis

The mice were euthanized with 100 mg/kg sodium pentobarbital, followed by cervical dislocation. The total lumbar part 3–5 of DRG, lumbar dorsal horn of SC, thalamus, SSC, and CB5-7 of the mice were excised to extract proteins. Tissues were placed on ice and stored at −80 °C until protein extraction. Protein extracts were prepared; then, 10% radioimmunoprecipitation (RIPA) lysis buffer assay (Fivephoton Biochemicals, San Diego, CA, USA, RIPA-50), 100 μL of a protease inhibitor (FC0070-0001, Bionovas, No. 194 Kingsdale Ave, Toronto, ON, Canada), and 100 μL of phosphatase inhibitor (FC0050-0001, Bionovas, No. 194 Kingsdale Ave, Toronto, ON, Canada) were added to the lysate. After homogenization, the mixture was centrifuged at 10,000 rpm for 10 min at 4 °C in an adjustable centrifuge. A total of 10 μL of extracted tissue sample was subjected to 8% or 12% SDS Tris-glycine gel electrophoresis, according to the protein size. Current from an electrophoresis power supply was used to run the gels in two sections: Section 1 for 40 min at 50 V and Section 2 for 1 h and 50 min at 100 V. A semidry transfer machine (Trans-Blot SD Cell, Hercules, CA, USA) was used to transfer the gels onto PVDF membranes at 15 V for 45 min, which were washed with phosphate-buffered saline Tween (PBST with 0.05% Tween 20) and blocked with bovine serum albumin for 30 min at 4 °C. Thereafter, the membranes were cultured with primary monoclonal antibodies in PBST with 1% BSA and incubated overnight at 4 °C. The following antibodies were used: GFAP (~50 kDa, 1:1000, cat no. AB5804, Merck KGaA, Darmstadt, Germany); Iba1 (~17 kDa, 1:1000, cat no. ACS-010, Alomone, Jerusalem, Israel); HMGB1 (∼28 kDa, 1:1000; cat no. SAB210867, Merck); S100B (∼10 kDa, 1: 1000; cat no. S2532, Merck); PD-1 (~55 kDa, 1:1000, cat no. 4-9969-82, Invitrogen, Waltham, MA, USA); TLR4 (~35 kDa, 1:1000, cat no. MABF2274, Merck); MyD88 (~35 kDa, 1:1000, cat no. AB16527, Merck); TRAF6 (~87 kDa, 1:1000, cat no. 23-059, Merck); and pNFκB (~65 kDa, 1:1000, cat no. 481408, Merck). Then, the membranes were incubated in a 1:5000 ratio mix of peroxidase-conjugated goat anti-rabbit antibody (Jackson Immuno Research Laboratory, West Grove, PA, USA) and goat anti-mouse antibody (Jackson Immuno Research Laboratory) for 2 h at 25 °C. Finally, we used an enhanced chemiluminescence substrate kit (PIERCE, Appleton, WI, USA) to visualize the protein bands on the membranes with LAS-3000 Fujifilm (Fuji Photo Film Co., Ltd., Tokyo, Japan). The image density levels of specific protein bands were quantified using NIH Image J 1.54h software (Bethesda, MD, USA). α-Tubulin (55 kDa) was used as the internal control. The percentage was calculated by dividing the value from the treatment group by that from the normal group.

### 2.5. Injection of Intracerebral Ventricles with PD-L1

Adult C57BL/6 female mice (*n* = 9) were selected for PD-L1 injection. After the initiation of FM in mice, PD-L1 (Abcam, Cambridge, UK, catalog: ab180058) was injected into i.c.v. at a dose of 5 μg under light isoflurane anesthesia (1%). PD-L1 was injected with a period of 3 min via a syringe pump (KD Scientific, Holliston, MA, USA). After injection, the cannula was retained for an additional 2 min to allow the PD-L1 solution to be wholly injected.

### 2.6. Statistical Analysis

We used the G*Power 3.1.9.7 statistic software program to determine the sample size. Statistical analysis was performed using the SPSS 21.0 statistical program. A Shapiro–Wilk test was performed to examine the normality of the results. All statistical data are presented as the mean ± standard error of the mean (SEM). Differences between groups were analyzed using an analysis of variance test (ANOVA), followed by Tukey’s post hoc test. *p* < 0.05 was considered statistically significant.

## 3. Results

### 3.1. EA or PD-L1 Injection Relieved Mechanical and Thermal Hyperalgesia in an Intermittent Cold Stress-Induced FM Mouse Model

**Figure 1:**
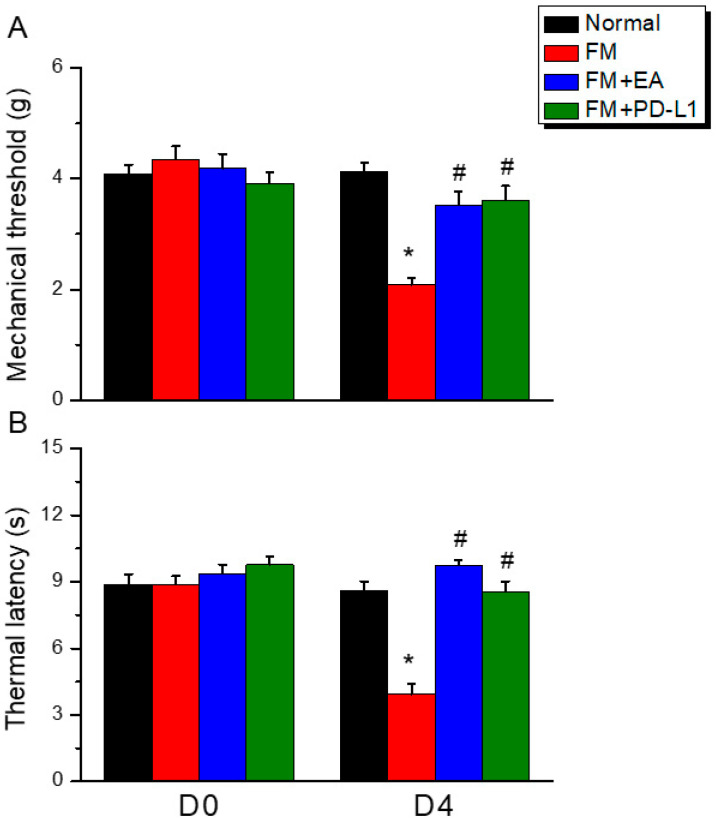
Electroacupuncture and PD-L1 i.c.v. inoculation reduced fibromyalgia pain after ICS. (**A**) Mechanical hyperalgesia verified using the von Frey test. (**B**) Thermal hyperalgesia determined using Hargraves’ test. Normal: normal mice; FM: fibromyalgia mice; FM + EA: fibromyalgia mice treated with EA; FM + PD-L1: fibromyalgia mice treated with PD-L1. * *p* < 0.05 vs. Normal. ^#^ *p* < 0.05 vs. FM group. *n* = 9.

To explore the role of EA and PD-L1 in fibromyalgia pain, we first generated a fibromyalgia pain model via ICS induction. Compared to normal mice (Figure 1A, black column, day 4: 4.12 ± 0.16 g, *n* = 9), the fibromyalgia mice showed a lower mechanical pain threshold (Figure 1A, red column, day 4: 2.08 ± 0.13 g, Tukey’s test, * *p* < 0.05, *n* = 9), indicating mechanical hyperalgesia. To confirm that EA had therapeutic effects on the fibromyalgia mice, we subjected them to 2 Hz EA. Our results demonstrated that the EA treatment successfully diminished mechanical hyperalgesia (Figure 1A, blue column, day 4: 3.53 ± 0.24 g, Tukey’s test, ^#^ *p* < 0.05, *n* = 9). In addition, i.c.v. PD-L1 injection showed an analgesic effect in the fibromyalgia mice (Figure 1A, green column, day 4: Tukey’s test, ^#^ *p* < 0.05, 3.61 ± 0.25 g, *n* = 9). As expected, the Hargreaves’ test showed significant thermal hyperalgesia in the fibromyalgia mice (Figure 1B, red column, day 4: Tukey’s test, * *p* < 0.05, 3.93 ± 0.45 g, *n* = 9); however, the 2 Hz EA treatment alleviated this (Figure 1B, blue column, day 4: Tukey’s test, ^#^ *p* < 0.05, 9.75 ± 0.23 g, *n* = 9). Furthermore, the mice subjected to i.c.v. PD-L1 injection presented a longer thermal latency, suggesting this treatment’s analgesic effect (Figure 1B, green column, day 4: Tukey’s test, ^#^ *p* < 0.05, 8.56 ± 0.45 g, *n* = 9).

### 3.2. Beneficial Effects of EA or PD-L1 on Fibromyalgia Pain Were Mediated via Microglia, Astrocytes, and PD-1 Pathway in the Mouse DRG

**Figure 2:**
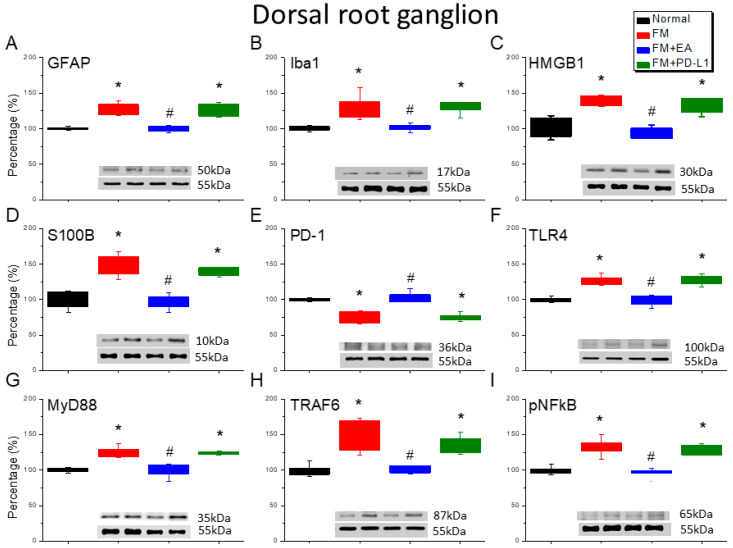
The expression levels of PD-1 were lower, and pain-related molecule levels increased at day 4 after fibromyalgia induction in the DRG in all groups. Western blot with four lanes of each protein: Normal, FM, FM + EA, and FM + PD-L1. (**A**) GFAP, (**B**) Iba1, (**C**) HMGB1, (**D**) S100B, (**E**) PD-1, (**F**) TLR4, (**G**) MyD88, (**H**) TRAF6, and (**I**) pNF-κB protein levels in Normal, FM, FM + EA, and FM + PD-L1. * *p* < 0.05 vs. Normal. ^#^ *p* < 0.05 vs. FM group. *n* = 6 mice per group. The percentage was calculated by dividing the value from the treatment group by that from the normal group.

The effects of EA or PD-L1 on microglia, astrocytes, and PD-1 receptor expression were evaluated 4 days after fibromyalgia induction. On day 4, microglia (Iba1) and astrocytes (GFAP) were significantly increased in the L3–L5 DRG compared with the normal mice (Figure 2A,B, Tukey’s test, * *p* < 0.05, *n* = 6). Iba1 or GFAP potentiation was alleviated by the EA treatment, but not with the i.c.v. PD-L1 injection. Next, we determined whether HMGB1 and S100B were involved in this fibromyalgia model and the effects of EA or PD-L1. Our results indicated that both HMGB1 and S100B were increased after fibromyalgia induction, and this was mitigated by EA (Figure 2C,D, Tukey’s test, * *p* < 0.05, *n* = 6) but not by PD-L1 injection. PD-1 expression in the DRG decreased in the fibromyalgia mice (Figure 2E, Tukey’s test, * *p* < 0.05, *n* = 6); the 2 Hz EA increased this again, but the PD-L1 injection did not. TLR4 was amplified in the fibromyalgia mice’s DRGs (Figure 2F, Tukey’s test, * *p* < 0.05, *n* = 6). However, this amplification was reduced upon EA administration on day 4 but not in the PD-L1-treated group. MyD88 was increased in the fibromyalgia mice’s DRGs compared with the normal group (Figure 2G, Tukey’s test, * *p* < 0.05, *n* = 6). The 2 Hz EA, but not the PD-L1 injection, significantly attenuated MyD88 overexpression. Next, we observed TRAF6 expression in the mice’s DRGs: this was greater in the fibromyalgia mice (Figure 2H, Tukey’s test, * *p* < 0.05, *n* = 6) and was inhibited by EA but not by PD-L1. Finally, pNFκB was detected in the DRGs of the normal mice and was overexpressed in the DRGs of the fibromyalgia mice (Figure 2I, Tukey’s test, * *p* < 0.05, *n* = 6). This increase was markedly reduced in the mice that received EA but not in those that received PD-L1.

### 3.3. EA and PD-L1 Treatment Reduced FM Pain in the Spinal Cord

**Figure 3:**
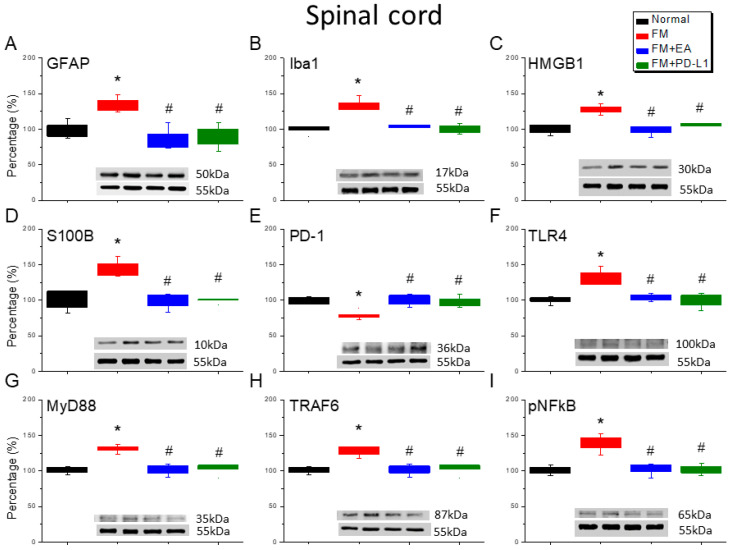
The expression levels of PD-1 were lower, and pain-related molecule levels increased at day 4 after fibromyalgia induction in the SC in all groups. Western blot with four lanes of each protein: Normal, FM, FM + EA, and FM + PD-L1. (**A**) GFAP, (**B**) Iba1, (**C**) HMGB1, (**D**) S100B, (**E**) PD-1, (**F**) TLR4, (**G**) MyD88, (**H**) TRAF6, and (**I**) pNF-κB protein levels in Normal, FM, FM + EA, and FM + PD-L1. * *p* < 0.05 vs. Normal. ^#^ *p* < 0.05 vs. FM group. *n* = 6 mice per group. The percentage was calculated by dividing the value from the treatment group by that from the normal group.

The ICS-induced fibromyalgia mice exhibited an increase in the number of microglia and astrocytes labeled with Iba1 and GFAP compared to the normal mice (Figure 3A,B, Tukey’s test, * *p* < 0.05, *n* = 6). In addition, 2 Hz EA treatment significantly decreased the number of those cells, with similar results also observed in mice receiving i.c.v. PD-L1 injection (Figure 3A,B, Tukey’s test, ^#^ *p* < 0.05, *n* = 6). HMGB1 and S100B expression was markedly increased in the lumbar SC of the fibromyalgia mice compared to that in the normal mice (Figure 3C,D, Tukey’s test, * *p* < 0.05, *n* = 6). In addition, this increase can be inhibited by 2 Hz EA treatment or PD-L1 injection, aligning with the behavioral results (Figure 3C,D, Tukey’s test, ^#^ *p* < 0.05, *n* = 6). We found lower PD-1 expression in the fibromyalgia mice (Figure 3E, Tukey’s test, * *p* < 0.05, *n* = 6), which could be increased using 2 Hz EA or PD-L1 (Figure 3E, Tukey’s test, ^#^ *p* < 0.05, *n* = 6). The TLR4 and MyD88 levels increased upon fibromyalgia induction (Figure 3F,G, Tukey’s test, * *p* < 0.05, *n* = 6), which was attenuated by the EA treatment and PD-L1 injection (Figure 3F,G, Tukey’s test, ^#^ *p* < 0.05, *n* = 6). The TRAF6 expression in the lumbar SC was increased in the fibromyalgia mice (Figure 3H, Tukey’s test, * *p* < 0.05, *n* = 6). This potentiation was alleviated by both the EA treatment and PD-L1 injection (Figure 3H, Tukey’s test, ^#^ *p* < 0.05, *n* = 6). Furthermore, we detected increased pNFκB levels (Figure 3I, Tukey’s test, * *p* < 0.05, *n* = 6) in the SC, which were notably reduced by the EA treatment and PD-L1 injection (Figure 3I, Tukey’s test, ^#^ *p* < 0.05, *n* = 6).

### 3.4. 2 Hz EA or PD-L1 Treatment Can Reverse Microglial/Astrocytic Activation and TLR4 Accumulation in the Thalamus of FM Mice

**Figure 4:**
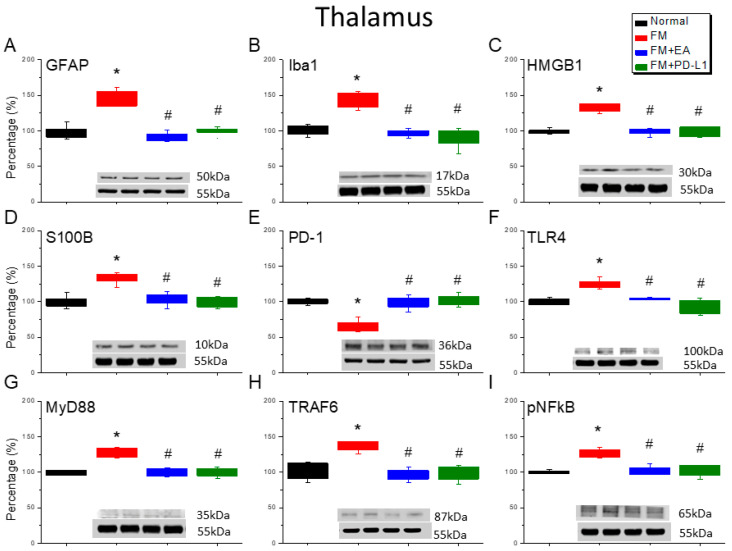
The expression levels of PD-1 were lower, and pain-related molecule levels increased at day 4 after fibromyalgia induction in the thalamus in all groups. Western blot with four lanes of each protein: Normal, FM, FM + EA, and FM + PD-L1. (**A**) GFAP, (**B**) Iba1, (**C**) HMGB1, (**D**) S100B, (**E**) PD-1, (**F**) TLR4, (**G**) MyD88, (**H**) TRAF6, and (**I**) pNF-κB protein levels in Normal, FM, FM + EA, and FM + PD-L1. * *p* < 0.05 vs. Normal. ^#^ *p* < 0.05 vs. FM group. *n* = 6 mice per group. The percentage was calculated by dividing the value from the treatment group by that from the normal group.

The accumulation of Iba1-positive microglia and GFAP-positive astrocytes was found in the thalamus of the ICS-induced fibromyalgia mice (Figure 4A,B, Tukey’s test, * *p* < 0.05, *n* = 6). However, after 4 days of ICS, the Iba1 and GFAP levels were attenuated by the 2 Hz EA management and PD-L1 injection (Figure 4A,B, Tukey’s test, ^#^ *p* < 0.05, *n* = 6). HMGB1 and S100B accumulation in the thalamus started at day 4 after ICS (Figure 4C,D, Tukey’s test, * *p* < 0.05, *n* = 6); however, these levels decreased in the mice subjected to 2 Hz EA or PD-L1 injection (Figure 4C,D, Tukey’s test, ^#^ *p* < 0.05, *n* = 6). Notably, a reduction in PD-1 expression was observed in the thalamus of the fibromyalgia mice (Figure 4E, Tukey’s test, * *p* < 0.05, *n* = 6). Likewise, 2 Hz EA and PD-L1 resulted in increased PD-1 expression (Figure 4A,B, Tukey’s test, ^#^ *p* < 0.05, *n* = 6). Furthermore, augmented levels of TLR4-MyD88-TRAF6 were detected in the thalamus of the fibromyalgia mice (Figure 4F–H, * *p* < 0.05, *n* = 6), which decreased with the 2 Hz EA and PD-L1 (Figure 4F–H, Tukey’s test, ^#^ *p* < 0.05, n = 6). The pNFκB expression in the thalamus of the fibromyalgia mice was higher than that in the normal mice (Figure 4I, Tukey’s test, * *p* < 0.05, *n* = 6). The 2 Hz EA treatment and PD-L1 injection reliably reversed this expression pattern (Figure 4I, Tukey’s test, ^#^ *p* < 0.05, *n* = 6).

### 3.5. EA or PD-L1 Treatment Reduced FM Pain by Inhibiting the Microglia/Astrocyte-TLR4 Pathway in the Somatosensory Cortex

**Figure 5:**
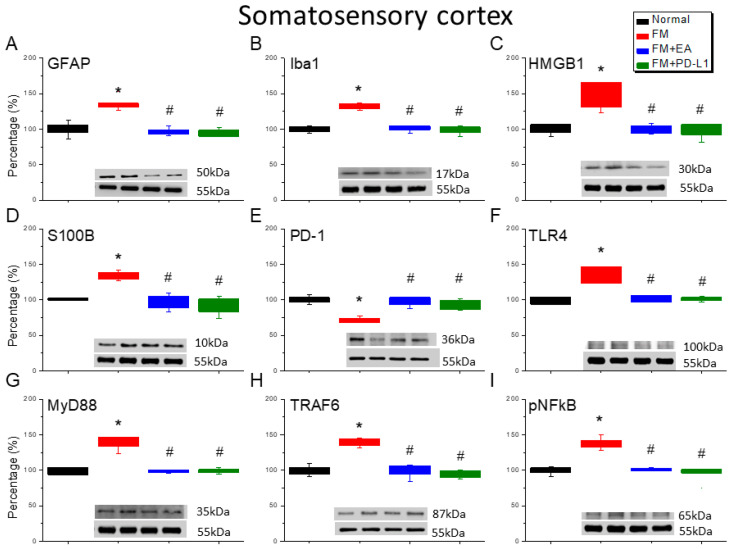
The expression levels of PD-1 were lower, and pain-related molecule levels increased at day 4 after fibromyalgia induction in the SSC in all groups. Western blot with four lanes of each protein: Normal, FM, FM + EA, and FM + PD-L1. (**A**) GFAP, (**B**) Iba1, (**C**) HMGB1, (**D**) S100B, (**E**) PD-1, (**F**) TLR4, (**G**) MyD88, (**H**) TRAF6, and (**I**) pNF-κB protein levels in Normal, FM, FM + EA, and FM + PD-L1. * *p* < 0.05 vs. Normal. ^#^ *p* < 0.05 vs. FM group. *n* = 6 mice per group. The percentage was calculated by dividing the value from the treatment group by that from the normal group.

Iba1 and GFAP expression was observed in the SSCs of the normal mice; the levels of these proteins were increased in the fibromyalgia mice (Figure 5A,B, Tukey’s test, * *p* < 0.05, *n* = 6), but the 2 Hz EA treatment significantly attenuated this effect (Figure 5A,B, Tukey’s test, ^#^ *p* < 0.05, *n* = 6), and a similar tendency was observed with PD-L1 injection (Figure 5A,B, Tukey’s test, ^#^ *p* < 0.05, *n* = 6). In addition, there were increased HMGB1 and S100B immuno-positive signals in the SSCs of the fibromyalgia mice compared to those in the normal mice (Figure 5C,D, Tukey’s test, * *p* < 0.05, *n* = 6). A significant decrease in HMGB1 and S100B immunolabeling was observed with 2 Hz EA and PD-L1 (Figure 5C,D, Tukey’s test, ^#^ *p* < 0.05, *n* = 6). Next, we detected increased PD-1 in the SSCs of the fibromyalgia mice (Figure 5E, Tukey’s test, * *p* < 0.05, *n* = 6); this was inhibited by 2 Hz EA and PD-L1 administration (Figure 5E, Tukey’s test, ^#^ *p* < 0.05, *n* = 6). In addition, the increased TLR4 and MyD88 concentrations in the SSCs of the fibromyalgia mice (Figure 5F,G, Tukey’s test, * *p* < 0.05, *n* = 6) were inhibited by 2 Hz EA or PD-L1 injection (Figure 5F,G, Tukey’s test, ^#^ *p* < 0.05, *n* = 6). Similarly, the TRAF6 levels increased after ICS induction (Figure 5H, Tukey’s test, * *p* < 0.05, *n* = 6) but decreased in the EA and PD-L1 groups (Figure 5H, Tukey’s test, ^#^ *p* < 0.05, *n* = 6). Augmented pNFκB levels were found in the fibromyalgia mice (Figure 5I, Tukey’s test, * *p* < 0.05, *n* = 6) and were further altered by 2 Hz EA and PD-L1 (Figure 5I, Tukey’s test, ^#^ *p* < 0.05, *n* = 6).

### 3.6. Cold Stress Induced Fibromyalgia Pain by Increasing the Microglia/Astrocyte-TLR4 Pathway in the CB5-7, an Effect Reversed by EA and PD-L1 Treatment

**Figure 6:**
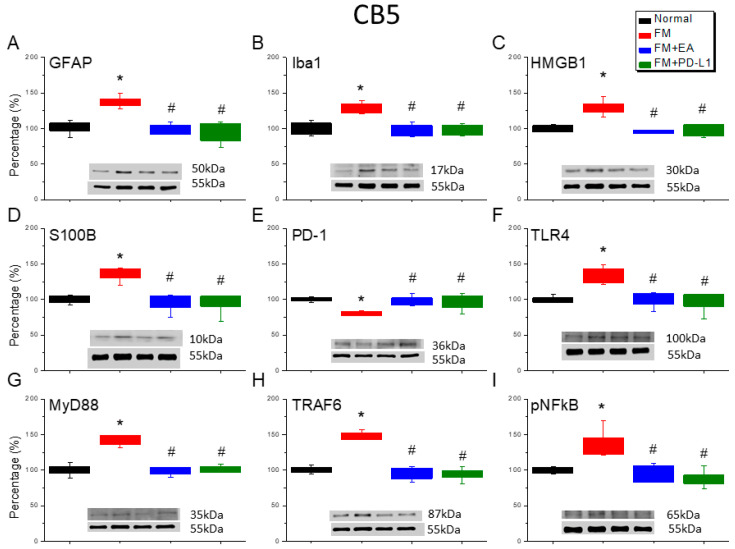
The expression levels of PD-1 were lower, and pain-related molecule levels increased at day 4 after fibromyalgia induction in the CB5 in all groups. Western blot with four lanes of each protein: Normal, FM, FM + EA, and FM + PD-L1. (**A**) GFAP, (**B**) Iba1, (**C**) HMGB1, (**D**) S100B, (**E**) PD-1, (**F**) TLR4, (**G**) MyD88, (**H**) TRAF6, and (**I**) pNF-κB protein levels in Normal, FM, FM + EA, and FM + PD-L1. * *p* < 0.05 vs. Normal. ^#^ *p* < 0.05 vs. FM group. *n* = 6 mice per group. The percentage was calculated by dividing the value from the treatment group by that from the normal group.

**Figure 7:**
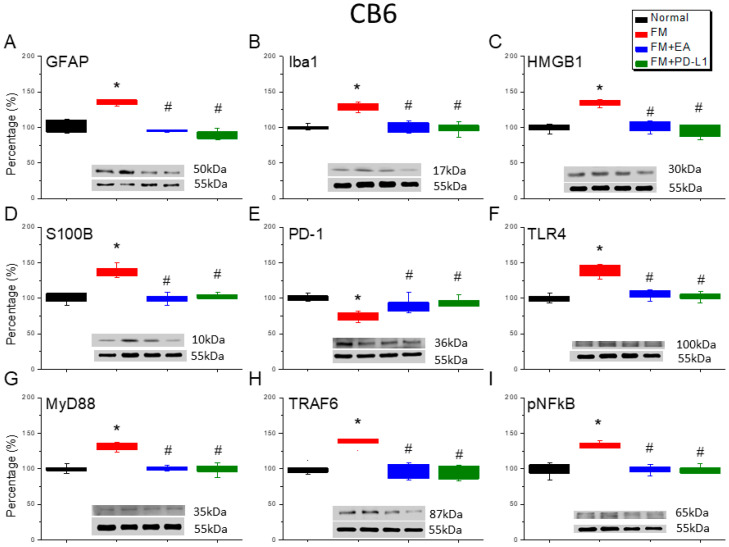
The expression levels of PD-1 were lower, and pain-related molecule levels increased at day 4 after fibromyalgia induction in the CB6 in all groups. Western blot with four lanes of each protein: Normal, FM, FM + EA, and FM + PD-L1. (**A**) GFAP, (**B**) Iba1, (**C**) HMGB1, (**D**) S100B, (**E**) PD-1, (**F**) TLR4, (**G**) MyD88, (**H**) TRAF6, and (**I**) pNF-κB protein levels in Normal, FM, FM + EA, and FM + PD-L1. * *p* < 0.05 vs. Normal. ^#^ *p* < 0.05 vs. FM group. *n* = 6 mice per group. The percentage was calculated by dividing the value from the treatment group by that from the normal group.

**Figure 8:**
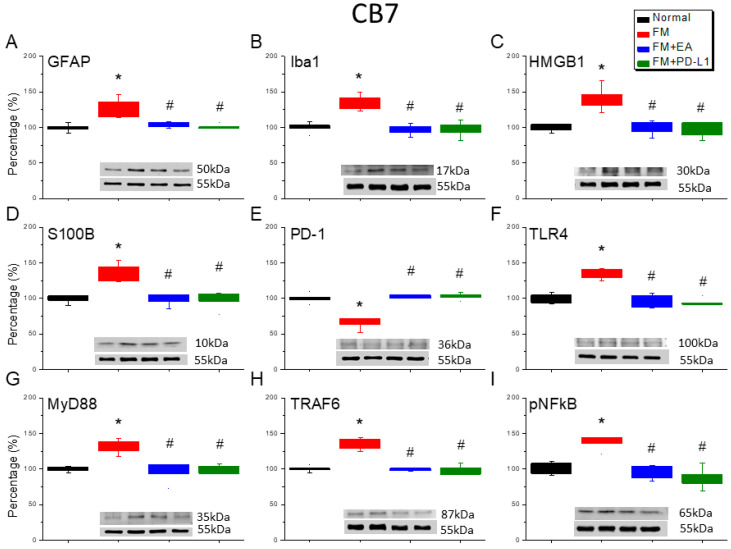
The expression levels of PD-1 were lower, and pain-related molecule levels increased at day 4 after fibromyalgia induction in the CB7 in all groups. Western blot with four lanes of each protein: Normal, FM, FM + EA, and FM + PD-L1. (**A**) GFAP, (**B**) Iba1, (**C**) HMGB1, (**D**) S100B, (**E**) PD-1, (**F**) TLR4, (**G**) MyD88, (**H**) TRAF6, and (**I**) pNF-κB protein levels in Normal, FM, FM + EA, and FM + PD-L1. * *p* < 0.05 vs. Normal. ^#^ *p* < 0.05 vs. FM group. *n* = 6 mice per group. The percentage was calculated by dividing the value from the treatment group by that from the normal group.

We detected a noteworthy increase in GFAP and Iba1 expression in the ICS-induced fibromyalgia pain mice (Figure 6A,B, Figure 7A,B and Figure 8A,B, Tukey’s test, * *p* < 0.05, *n* = 6). The potentiated effect was reversed by 2 Hz EA and PD-L1 injection (Figure 6A,B, Figure 7A,B and Figure 8A,B, Tukey’s test, ^#^ *p* < 0.05, *n* = 6). ICS also augmented HMGB1 and S100B concentrations in the mice’s CB 5–7 regions (Figure 6C,D, Figure 7C,D and Figure 8C,D, Tukey’s test, * *p* < 0.05, *n* = 6). Such intensification was then reduced by 2 Hz EA and PD-L1 injection (Figure 6C,D, Figure 7C,D and Figure 8C,D, Tukey’s test, ^#^ *p* < 0.05, *n* = 6). In addition, the PD-1 receptor was reduced in the mice’s CB5-7 regions after ICS (Figure 6E, Figure 7E and Figure 8E, Tukey’s test, * *p* < 0.05, *n* = 6). These phenomena were also diminished by the 2 Hz EA and PD-L1 treatment (Figure 6E, Figure 7E and Figure 8E, Tukey’s test, ^#^ *p* < 0.05, *n* = 6). Regarding TLR4 cascades, our results indicated comparable changes in the TLR4, MyD88, and TRAF6 protein concentrations in the fibromyalgia mice with those in the normal mice (Figure 6F–H, Figure 7F–H and Figure 8F–H, Tukey’s test, * *p* < 0.05, *n* = 6). The increased protein levels were considerably reduced by 2 Hz EA and *PD-L1* injection. The results for pNFκB were similar (Figure 6I, Figure 7I and Figure 8I, *n* = 6).

## 4. Discussion

In this study, we identified an effect of EA on fibromyalgia pain mediated through PD-L1/PD-1 signaling. The fibromyalgia mouse models exhibited mechanical and thermal hyperalgesia, with nociception attenuated by 2 Hz EA or PD-L1 injection. We found that the PD-1 receptor was reduced in the peripheral DRG as well as in the central SC, thalamus, SSC, and CB5-7 of the mice. We also confirmed that microglia/astrocytes and the TLR4 pathway were stimulated after FM induction. These trends were reversed by 2 Hz EA or PD-L1 injection. Our findings suggest that the administration of these treatments, due to their effect mediated through PD-1, could be a beneficial therapy for fibromyalgia. All results are displayed as standard error of mean (S.E.M) (Table S1).

Growing scientific evidence indicates that the immune checkpoint PD-L1 could be stimulated in cancer cells to inhibit T-cell function through activating the PD-1 receptor. Less is known about how the PD-1 signaling pathway regulates pain signaling, especially in terms of neuromodulation. One study reported that healthy DRG tissue could release PD-L1 to notably inhibit either acute or chronic pain. Local PD-L1 injection reliably induces antinociception in healthy mice through PD-1 activation. In addition, PD-L1 inhibition via neutralization of the PD-1 receptor initiates considerable mechanical allodynia, suggesting its role in pain control. Our results demonstrated that EA efficiently relieved nociception and led to low PD-1 levels in fibromyalgia mice. Deletion of *Pd1* results in thermal and mechanical hyperalgesia. The released PD-L1 binds to the PD-1 receptor of nociceptive DRG neurons, resulting in the phosphorylation of Src homology region 2 domain-containing phosphatase 1 (SHP-1), which can then suppress sodium channels and induce neuronal hyperpolarization by triggering potassium channels [28]. TLRs expressed in primary nociceptive neurons are important modulators of the immune system; they also regulate pain sensation via modulating ion channels; however, their relationship with PD-L1 has not been elucidated. Our results indicate that PD-L1 injection can attenuate fibromyalgia in mice: nociceptive-associated TLR4 was increased in fibromyalgia mice and could be diminished by PD-L1 injection.

Wang et al. reported that the immunotherapy nivolumab, a monoclonal antibody targeting PD-1, can suppress tumors. They utilized a genetic knockout of the PD-1 receptor (*Pd1*^−/−^) to demonstrate its ability to defend against lung cancer cell-induced bone destruction. Mice lacking the PD-1 receptor showed higher nociceptive responses than healthy mice. In addition, this study determined that PD-L1 and chemokine ligand 2 (CCL2) were simultaneously increased in the tumor microenvironment. Cancer cells also exhibited increased chemokine receptor 2 (CCR2) expression in primary sensory neurons. CCR2 antagonism also led to significant attenuation of bone cancer pain [29]. SC injury (SCI) causes motor disability and neuropathic pain, which is difficult to treat. After SCI, PD-L1 increased in the microglia present at the epicenter of the injury site. Mice without PD-L1 showed more serious neuropathic pain due to increased polarization of M1-like microglia compared with that in normal mice. Furthermore, PD-L1 attenuated neuropathic pain after SCI by inhibiting pp38 and pERK1/2 [30]. Our findings showed that EA and PD-L1 injection can mitigate mice fibromyalgia by decreasing TLR4 and downstream molecules such as MyD88, TRAF6, and pNF-κB. Shi et al. observed PD-L1 and PD-1 immuno-positive signals in normal trigeminal ganglia neurons. They also determined that PD-L1 and PD-1 mRNA and protein levels were considerably increased in an acute nitroglycerin-induced mouse migraine model. Furthermore, they indicated that PD-1 receptor inhibition potentiated acute nitroglycerin-induced migraines. This phenomenon is also seen with amplified calcitonin gene-related peptide, IL-1β, IL-6, and TNF-α expression in the trigeminal ganglia of mouse models of neuropathic pain [31]. Our findings indicated that EA and PD-L1 could reliably alleviate FM pain through the PD-1 receptor to attenuate the microglia/astrocyte signaling pathway.

After a stroke, patients often experience thalamic pain, which is a neuropathic pain syndrome; an increase in cases of this type of neuralgia has been observed. A recent study declared that collagenase IV injected into the right thalamus of mice induced thalamic pain, which could then be relieved by dexmedetomidine (a selective α2 adrenergic receptor agonist). The increased levels of Iba1, GFAP, and TLR4/NF-κB signaling in the thalamic pain mouse models were reversed by intraperitoneal injection of dexmedetomidine [32]. Research into the development of drugs for neuropathic pain is increasingly targeting ion channels in order to modulate pain sensation and management and signal transduction. Transient receptor potential channel melastatin 2 (TRPM2) has been reported to adjust the Ca^2+^ concentration and has a crucial effect on pain signaling, osmosis, and temperature sensing. A recent study indicated that TLR4 is one of the main receptors that respond to inflammation; its activation resulted in higher oxidative stress and expression of inflammatory cytokines such as IL-1β, IL-6, and TNF-α. These potentiated molecules can then activate TRPM2, which results in excessive influx of Ca^2+^, inducing neuronal damage [33]. Converting M1 cells and their microglial phenotypes, associated with increased proinflammatory factors, into M2 cells, which have neuroprotective effects, was reported as an encouraging approach for treating acute and chronic pain. A recent article declared that the anti-inflammatory dietary flavonoid kaempferol had an analgesic effect in a mouse model of chronic constriction injury-induced neuropathic pain. Their data indicated that kaempferol reliably relieved neuropathic pain, accompanied by reduced proinflammatory cytokine production, by reducing neuropathic pain-induced TLR4/NF-κB overexpression in the SC. Kaempferol can attenuate neuropathic pain by promoting microglial polarization from the M1 to the M2 phenotype [34]. A recent article mentioned that pulsed laser acupuncture, performed using an individualized protocol, significantly diminished pain according to the pain numerical scale, generalized pain index, and symptom severity scale [35]. Minakawa et al. indicated that a combination of electroacupuncture with scalp acupuncture efficiently relieved pain and decreased pregabalin use in people with fibromyalgia [36]. Wu et al. showed that electroacupuncture significantly increased the expression levels of PD-L1/PD-1 and decreased the levels of pERK, pp38, pJNK, and p-NFκB pathways in a spinal nerve ligation pain model [37]. We suggest EA as a potential therapy that activates the PD-1 receptor signaling pathway and thus could serve as a practical treatment for people with fibromyalgia.

## 5. Conclusions

**Figure 9:**
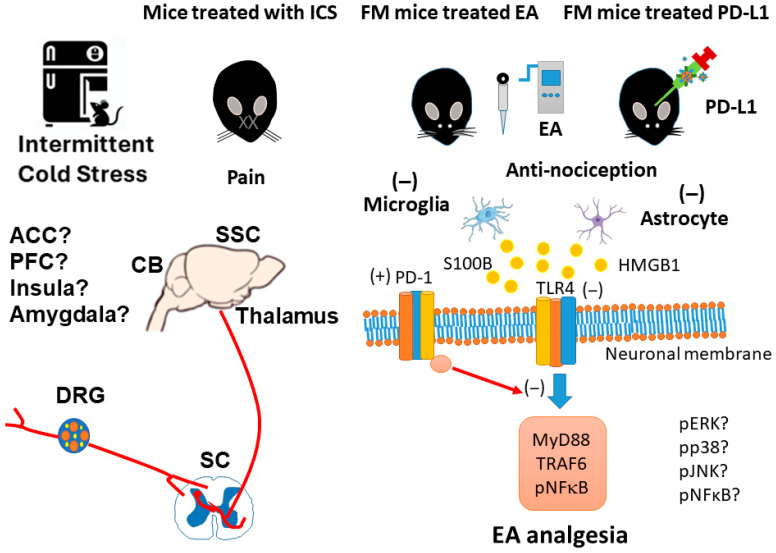
The effect of EA on the PD-1 signaling pathway in the fibromyalgia mouse model. Abbreviations: EA = electroacupuncture; TLR4 = Toll-like receptor 4; PD-L1 = programmed cell death ligand 1; PD-1 = programmed cell death protein 1; MyD88 = myeloid differentiation primary response 88; TRAF6 = TNF Receptor-Associated Factor 6; pNF-kB = phosphorylated nuclear factor kappa-light-chain-enhancer of activated B cells.

We found that mechanical and thermal pain in mice with ICS-induced fibromyalgia was reversed by 2 Hz EA and PD-L1 injection. Further, levels of microglia/astrocytes and also HMGB1 and S100B were increased, whereas PD-1 was decreased, in the DRG, SC, thalamus, SSC, and CB5-7 of fibromyalgia mice. Also, the expression of nociceptive TLR4 and its related downstream elements increased after fibromyalgia induction, which was then repressed by EA or PD-L1 injection in all of the tested regions except for the DRG, where PD-L1 injection could not reverse changes in expression, suggesting it is ineffective here. Our results indicate that EA’s effect on the PD-L1/PD1 pathway might be the key to its potential as a therapeutic target for fibromyalgia (Figure 9). Our key discoveries are that FM pain was associated with the PD-L1 signaling pathway, EA significantly reduced FM pain through increasing PD-1 receptors, and i.c.v. injection of PD-L1 suggests the PD-1 receptor has precise mechanisms that lead to analgesic effects. A limitation of the present study was that we only injected the FM mice with PD-L1. In the future, PD-1 knockout models should also be injected with PD-L1. The current study also had a small sample size; in future studies, a larger group is needed for the detection of larger effect sizes, with a power of at least 60%. Also, other animal models that exhibit greater similarity with clinical FM need to be studied in the future. We have to perform immunofluorescence staining at specific regions of DRG, SC, thalamus, SSC, and CB5-7 of fibromyalgia mice to determine which regions are involved in pain transmission. We will also conduct experiments to examine the PD-L1/PD1 pathway in pain-associated regions such as the anterior cingulate cortex, prefrontal cortex, insula, and amygdala in the future. We suggest that future research should implement these recommendations so that EA can be established as an FM treatment.

## Data Availability

The original contributions presented in this study are included in the article. Further inquiries can be directed to the corresponding authors.

## Supplementary Materials

The following supporting information can be downloaded at https://www.mdpi.com/article/10.3390/brainsci15090976/s1, Table S1: Evaluation of protein percentages (%) in 7 regions across four groups.

## Abbreviations

The following abbreviations are used in this manuscript:

ACC	Anterior cingulate cortex
CB	Cerebellum
CCL2	Chemokine ligand 2
CCR2	Chemokine receptor 2
DRG	Dorsal root ganglion
EA	Electroacupuncture
GFAP	Glial fibrillary acidic protein
HMGB1	High-mobility group protein B1
Iba1	Ionized calcium-binding adaptor molecule 1
ICS	Intermittent cold stress
IFN-γ	Interferon gamma
IL	Interleukin
MyD88	Myeloid differentiation primary response 88
PD-1	Programmed cell death protein 1
PD-L1	Programmed cell death ligand 1
pERK	Phosphorylated extracellular signal-regulated kinase
PFC	Prefrontal cortex
pNF-kB	Phosphorylated nuclear factor kappa-light-chain-enhancer of activated B cells.
S100B	S100 calcium-binding protein B
SC	Spinal cord
SCI	Spinal cord injury
SHP-1	Src homology region 2 domain-containing phosphatase 1
SSC	Somatosensory cortex
TLR4	Toll-like receptor 4
TNF-α	Tumor necrosis factor alpha
TRAF6	TNF Receptor Associated Factor 6
TRPM2	Transient receptor potential channel melastatin 2
