# Supplementary material for: Electroacupuncture Attenuates Fibromyalgia Pain Through Increased PD-1 Expression in Female Mice
Source: Brain Sci. 2025 Sep 11;15(9):976. doi: 10.3390/brainsci15090976 (PMC12467811; doi:10.3390/brainsci15090976)
Supplement: Supplementary file 1 [file brainsci-15-00976-s001.zip › brainsci-3808833-supplementary.pdf]

Table S1 Evaluation of protein percentages (%) in 7 regions across four groups. The normal group was set as 100% and served as a reference. Statistical variances were examined using an analysis of variance test, followed by a post hoc Tukey's test.

*signifies a significant difference, compared to the normal group ($p < 0.05$); #means a significant difference, compared to FM group ($p < 0.05$).

Region		GFAP		Iba1		HMGB1		S100B		PD-1		TLR4		MyD88		TRAF6		pNFkB	
		Mean	SE	Mean	SE	Mean	SE	Mean	SE	Mean	SE	Mean	SE	Mean	SE	Mean	SE	Mean	SE
DRG	Normal	100.0	0.9	100.0	1.3	100.0	6.8	100.0	4.9	100.0	1.0	100.0	1.4	100.0	1.3	100.0	3.2	100.0	2.2
	*FM	127.1	3.6	130.5	6.6	139.8	2.9	145.6	6.4	75.6	3.2	126.7	2.6	125.2	3.0	146.5	8.5	133.1	4.7
	#FM+EA	100.6	1.8	101.5	2.1	90.2	8.6	96.4	3.9	102.3	3.0	99.3	3.1	99.5	3.8	100.0	2.0	96.5	2.6
	#FM+PD-L1	125.0	3.7	136.6	9.6	138.2	9.1	145.4	7.3	74.3	2.1	126.2	2.9	124.1	0.8	135.4	5.1	130.0	2.9
SC	Normal	100.0	4.2	100.0	2.2	100.0	2.3	100.0	5.3	100.0	1.9	100.0	1.8	100.0	1.9	100.0	1.9	100.0	2.4
	*FM	133.2	3.9	134.3	3.0	127.6	2.3	144.9	4.4	78.7	2.3	131.5	4.4	131.4	2.0	128.0	2.8	139.6	4.4
	#FM+EA	87.8	5.3	102.3	2.2	98.7	2.4	100.1	4.2	100.1	3.0	103.9	1.9	101.7	2.8	101.7	2.8	101.7	2.9
	#FM+PD-L1	89.3	6.0	100.5	2.6	111.1	5.2	101.5	2.4	97.6	2.8	100.6	3.8	102.9	2.8	102.9	2.8	101.8	2.6
Thalamus	Normal	100.0	4.3	100.0	2.9	100.0	3.4	100.0	1.2	100.0	1.7	100.0	1.8	100.0	1.4	100.0	4.7	100.0	1.0
	*FM	144.8	5.6	143.7	4.5	131.4	3.0	131.5	2.3	65.5	3.4	123.7	2.7	126.5	2.7	136.1	3.0	127.0	2.5
	#FM+EA	92.1	3.1	96.6	2.1	102.2	3.5	98.9	1.9	98.7	3.5	103.0	1.6	99.1	2.2	96.0	3.3	102.1	2.5
	#FM+PD-L1	99.7	3.0	89.8	5.4	98.8	3.0	99.4	2.7	101.9	3.1	94.1	4.3	100.6	2.7	97.0	4.1	100.8	3.3
SSC	Normal	100.0	4.5	100.0	1.7	100.0	3.0	100.0	1.6	100.0	2.0	100.0	2.0	100.0	2.0	100.0	2.8	100.0	2.2
	*FM	136.1	4.4	132.4	1.7	149.3	6.9	133.8	2.3	71.9	1.4	135.9	4.2	137.6	3.5	139.1	2.3	138.3	3.1
	#FM+EA	97.2	2.5	101.3	1.7	98.4	2.7	98.4	4.1	97.9	2.7	100.6	1.8	98.9	1.5	99.1	3.6	97.1	3.7
	#FM+PD-L1	95.8	2.6	99.3	2.4	96.8	3.8	92.4	4.8	93.0	2.8	100.8	1.2	99.1	1.2	94.3	2.0	96.9	4.8

Region		GFAP		Iba1		HMGB1		S100B		PD-1		TLR4		MyD88		TRAF6		pNFkB	
		Mean	SE	Mean	SE	Mean	SE	Mean	SE	Mean	SE	Mean	SE	Mean	SE	Mean	SE	Mean	SE
CB5	Normal	100.0	3.5	100.0	3.5	100.0	2.0	100.0	2.2	100.0	1.2	100.0	1.8	100.0	3.1	100.0	2.0	100.0	1.8
	*FM	137.8	3.2	129.4	2.9	130.7	4.1	134.7	3.8	76.8	3.9	132.9	4.7	145.5	5.4	144.6	4.7	135.4	7.7
	[#]FM+EA	96.7	6.2	96.2	3.6	97.1	1.8	97.4	5.1	98.4	2.7	99.5	4.1	98.9	2.3	96.5	3.7	94.0	4.7
	[#]FM+PD-L1	95.9	5.8	96.7	3.1	95.9	3.2	94.0	5.5	94.7	4.4	96.0	5.5	97.3	4.3	94.1	3.4	86.6	4.5
CB6	Normal	100.0	3.5	100.0	1.4	100.4	2.2	100.0	2.8	100.0	1.9	100.0	2.1	100.0	1.9	100.0	2.8	100.0	3.7
	*FM	139.1	4.7	129.6	2.5	134.5	1.8	137.8	3.1	74.0	2.9	137.1	3.5	131.4	2.2	138.8	3.3	133.0	1.8
	[#]FM+EA	96.5	1.9	100.6	2.9	102.5	3.2	99.9	2.7	91.4	4.3	105.3	2.6	100.8	1.3	96.2	4.8	98.1	2.3
	[#]FM+PD-L1	91.1	2.6	98.4	3.1	94.8	3.6	101.0	3.7	92.0	4.0	102.9	2.4	99.9	2.9	96.0	3.9	95.4	5.3
CB7	Normal	100.0	2.1	100.0	2.7	100.0	1.9	100.0	3.2	100.0	2.4	100.0	2.6	100.0	1.3	100.0	1.5	100.0	3.2
	*FM	127.3	5.0	135.4	4.0	141.6	5.9	136.7	4.8	66.3	3.1	133.8	2.7	132.8	3.8	135.9	3.2	142.1	6.3
	[#]FM+EA	103.1	1.5	97.1	2.7	100.6	3.8	98.5	3.0	100.5	3.1	96.7	3.5	94.4	5.0	100.4	1.7	94.0	3.5
	[#]FM+PD-L1	100.1	1.8	97.7	4.1	95.6	4.1	98.1	4.4	102.5	1.6	92.0	3.0	95.9	4.5	98.2	2.5	86.1	5.3
